# Multilocus variable-number tandem repeat analysis for molecular typing and phylogenetic analysis of *Shigella flexneri*

**DOI:** 10.1186/1471-2180-9-278

**Published:** 2009-12-31

**Authors:** You-Wun Wang, Haruo Watanabe, Dac Cam Phung, Sheng Kai Tung, Yeong-Sheng Lee, Jun Terajima, Shiu-Yun Liang, Chien-Shun Chiou

**Affiliations:** 1 The Central Region Laboratory, Center for Research and Diagnostics, Centers for Disease Control, Taichung, Taiwan; 2 National Institute of Infectious Diseases, Tokyo, Japan; 3 Division of Enteric Infections, National Institute of Hygiene and Epidemiology, Vietnam; 4 The Sixth Branch Office, Centers for Disease Control, Hualien, Taiwan; 5 Institute of Medicine, Chung Shan Medical University, Taichung, Taiwan

## Abstract

**Background:**

*Shigella flexneri *is one of the causative agents of shigellosis, a major cause of childhood mortality in developing countries. Multilocus variable-number tandem repeat (VNTR) analysis (MLVA) is a prominent subtyping method to resolve closely related bacterial isolates for investigation of disease outbreaks and provide information for establishing phylogenetic patterns among isolates. The present study aimed to develop an MLVA method for *S. flexneri *and the VNTR loci identified were tested on 242 *S. flexneri *isolates to evaluate their variability in various serotypes. The isolates were also analyzed by pulsed-field gel electrophoresis (PFGE) to compare the discriminatory power and to evaluate the usefulness of MLVA as a tool for phylogenetic analysis of *S. flexneri*.

**Results:**

Thirty-six VNTR loci were identified by exploring the repeat sequence loci in genomic sequences of *Shigella *species and by testing the loci on nine isolates of different subserotypes. The VNTR loci in different serotype groups differed greatly in their variability. The discriminatory power of an MLVA assay based on four most variable VNTR loci was higher, though not significantly, than PFGE for the total isolates, a panel of 2a isolates, which were relatively diverse, and a panel of 4a/Y isolates, which were closely-related. Phylogenetic groupings based on PFGE patterns and MLVA profiles were considerably concordant. The genetic relationships among the isolates were correlated with serotypes. The phylogenetic trees constructed using PFGE patterns and MLVA profiles presented two distinct clusters for the isolates of serotype 3 and one distinct cluster for each of the serotype groups, 1a/1b/NT, 2a/2b/X/NT, 4a/Y, and 6. Isolates that had different serotypes but had closer genetic relatedness than those with the same serotype were observed between serotype Y and subserotype 4a, serotype X and subserotype 2b, subserotype 1a and 1b, and subserotype 3a and 3b.

**Conclusions:**

The 36 VNTR loci identified exhibited considerably different degrees of variability among *S. flexneri *serotype groups. VNTR locus could be highly variable in a serotype but invariable in others. MLVA assay based on four highly variable loci could display a comparable resolving power to PFGE in discriminating isolates. MLVA is also a prominent molecular tool for phylogenetic analysis of *S. flexneri*; the resulting data are beneficial to establish clear clonal patterns among different serotype groups and to discern clonal groups among isolates within the same serotype. As highly variable VNTR loci could be serotype-specific, a common MLVA protocol that consists of only a small set of loci, for example four to eight loci, and that provides high resolving power to all *S. flexneri *serotypes may not be obtainable.

## Background

*Shigella flexneri*, as well as *S. dysenteriae*, *S. boydii*, and *S. sonnei*, are the causative agents of shigellosis, an acute diarrheal disease common in developing countries. The annual number of shigellosis cases throughout the world has been estimated to be 164.7 million, of which 163.2 million were in developing countries, with 1.1 million deaths, and 1.5 million in industrialized countries [1]. *S. flexneri *is the predominant species in developing countries and the second most common in industrialized countries [1,2]. *S. flexneri *comprises eight serotypes, 1, 2, 3, 4, 5, 6, X, and Y, with at least 12 subserotypes, 1a, 1b, 1c, 2a, 2b, 3a, 3b, 4a, 4b, 4c, 5a, and 5b [3,4], of which 2a is the most prevalent subserotype in the world [1]. The X and Y serotypes can be derived from some subserotypes of serotypes 1, 2, 3, and 4, such as 2b, 4c and 5b for serotype X and 1a, 2a, 4a and 5a for serotype Y, by losing the type factor antigens [3-5]. Multilocus sequencing typing (MLST) analysis has revealed that the *S. flexneri *serotypes 1-5, X and Y are evolutionarily more related than serotype 6 [6].

A variety of molecular typing tools have been developed to access genetic relatedness among bacterial isolates. In general, molecular markers with low variability can be used to establish phylogenetic relationships among bacterial isolates evolved over longer time spans, and highly variable markers are more useful to resolve closely related isolates for the purposes of outbreak investigation and disease surveillance. MLST, one of these molecular tools, is a sequence-based method that has been successfully applied to establish phylogenetic structure for some bacterial pathogens, such as *Neisseria meningitidis *and *Streptococcus pneumoniae *[7]. However, to public health laboratories, MLST is not sufficiently discriminative in distinguishing closely related isolates for the epidemiological investigation of clusters of infection. In contrast, pulsed-field gel electrophoresis (PFGE) is highly discriminatory for many bacterial pathogens and has been adopted as the standard typing method by an international molecular subtyping network, PulseNet International, for foodborne disease surveillance [8]. Although this method has been proven by the PulseNet laboratories to be a powerful tool for the routine subtyping of some foodborne bacterial pathogens in detecting clusters of infection, PFGE is occasionally not discriminatory enough in distinguishing some epidemiologically unrelated *S. sonnei *isolates. In total, PFGE is suitable to resolve closely related isolates but not an appropriate tool for establishing phylogenetic relationships between bacterial isolates that have evolved over a longer time span.

Multilocus variable-number tandem repeat (VNTR) analysis (MLVA) is prominent typing tool which has been developed for a variety of bacterial pathogens [9-13]. This method is based on variation in the number of repeats at multiple VNTR loci, which can be highly variable or relatively stable. Study has demonstrated that MLVA based on four to eight highly variable VNTR loci can exhibit a discriminatory power parallel to or higher than PFGE [14] and that a method based on combined loci with different variability values can be applied to establish phylogenetic relationships among strains with different evolutionary timescales [15]. In the present study, we aim to develop and evaluate a MLVA method for fine typing and phylogenetic analysis of *S. flexneri *isolates.

## Results

### VNTR loci

In total, 36 VNTR loci were identified after testing 67 selected tandem repeat loci on nine *S. flexneri *isolates of various subserotypes. The locations, copy number, and sizes of amplicons for the 36 VNTRs in *S. flexneri *sequenced strains 301, 2457T and 8401 and the gene or encoding protein involved are listed in Table S1 (Additional file 1). Fifteen of the loci are located within pseudogenes. The 36 VNTR markers were analyzed on 242 *S. flexneri *isolates of various serotypes and subserotypes. The resulting data revealed that 28 of the 36 loci exhibited 100% typability (Table 1). Four loci (SF1, SF6, SF23 and SF26) had a low typability rate that was primarily attributed to the absence of PCR amplicon from 103 4a/Y isolates, which had common origin. There were, respectively, 9, 26, 19, 13 and 3 polymorphic loci detected in serotype groups 1a/1b/NT (non-typable serotype), 2a/2b/X/NT, 3a/3b, 4a/4b/Y and 6. There were three loci polymorphic in four of the five serotype groups, nine in three serotype groups, ten in two serotype groups and eleven in one serotype group. Three loci (SF20, SF28 and SF35) were not polymorphic in any of the five serotype groups. Ten loci bore alleles with a high copy number (≧ 6) of repeats (Table 2). Of the 10 loci, one locus (SF3) was polymorphic in four serotype groups, 2 in three serotype groups, 4 in two serotype groups and 3 in only one serotype group. Of the 10 VNTR loci, one that had low variability in the serotype groups usually bore an allele with a low copy number of repeats. SF12 was an exception; it had no polymorphism in the serotype groups 1a/1b/NT and 2a/2b/X/NT, but it contained an allele with a high number (eight copies) of repeats.

**Table 1:** Allelic diversity of VNTR loci in various serotype groups.

VNTR locus (alias)^a^	Range of copy number^b^	Typability^c ^(%)	Allele diversity for serotype group:					
			
			1a/1b/NT (n = 12)	2a/2b/X/NT (106)	3a/3b (n = 12)	4a/4b/Y (110)	6 (n = 2)	Total (n = 242)
SF1	N, 1-4	49.6	0.50	0.02	0.64	0.02	0.00	0.55
SF2 (ms09)	1-3, 283	100.0	0.15	0.04	0.28	0.31	0.00	0.52
SF3 (O157-11)	2-18	100.0	0.75	0.78	0.72	0.60	0.00	0.77
SF4	1-12	100.0	0.65	0.69	0.65	0.00	0.00	0.70
SF5	N, 1-3, 155, 161	99.2	0.00	0.16	0.57	0.00	0.00	0.62
SF6	N, 2-19	54.1	0.61	0.87	0.00	0.02	0.00	0.76
SF7	2-12	100.0	0.00	0.66	0.00	0.00	0.50	0.63
SF8 (ms22)	2-8	100.0	0.00	0.57	0.00	0.00	0.00	0.65
SF9	1-9	100.0	0.57	0.63	0.00	0.07	0.00	0.60
SF10	1-6	100.0	0.00	0.32	0.00	0.02	0.00	0.61
SF11 (ms25)	1-3	100.0	0.00	0.48	0.61	0.00	0.00	0.62
SF12 (ms07)	2-8, 389	100.0	0.00	0.00	0.50	0.05	0.00	0.54
SF13	1-3	100.0	0.00	0.39	0.00	0.00	0.00	0.44
SF14	N, 1-3	94.2	0.15	0.04	0.40	0.00	0.00	0.56
SF15	1-2	100.0	0.00	0.04	0.00	0.00	0.00	0.49
SF16	1-2	100.0	0.00	0.00	0.50	0.00	0.00	0.50
SF17	1-2	100.0	0.00	0.42	0.00	0.02	0.00	0.43
SF19	1-2	100.0	0.00	0.00	0.50	0.04	0.00	0.50
SF20	2-3	100.0	0.00	0.00	0.00	0.00	0.00	0.02
SF21	N, 1-2	97.1	0.00	0.07	0.57	0.00	0.00	0.22
SF22 (ms21)	1-4	100.0	0.00	0.11	0.00	0.07	0.00	0.54
SF23	N, 2-3	54.5	0.00	0.00	0.15	0.04	0.50	0.55
SF24	3-4, 241, 258	100.0	0.00	0.02	0.50	0.02	0.00	0.53
SF25	2-11	100.0	0.00	0.00	0.79	0.02	0.00	0.10
SF26	N, 1-3	47.9	0.44	0.20	0.28	0.00	0.00	0.57
SF27	2-4, 241	100.0	0.40	0.48	0.50	0.00	0.00	0.62
SF28	1-2	100.0	0.00	0.00	0.00	0.00	0.00	0.50
SF29	3-4	100.0	0.00	0.00	0.49	0.00	0.00	0.50
SF30	2-3	100.0	0.00	0.02	0.00	0.00	0.00	0.49
SF31	2-11	100.0	0.00	0.00	0.63	0.00	0.00	0.05
SF32	1-2	100.0	0.00	0.02	0.00	0.00	0.00	0.49
SF33 (ms06)	2-4	100.0	0.00	0.11	0.00	0.00	0.00	0.15
SF34	1-3	100.0	0.00	0.48	0.00	0.00	0.00	0.49
SF35	2-3	100.0	0.00	0.00	0.00	0.00	0.00	0.50
SF36	N, 1-2	97.1	0.00	0.02	0.57	0.00	0.50	0.53
SF37	1-2	100.0	0.00	0.02	0.00	0.00	0.00	0.49

^a^Alias loci described by Gorgé et al. [21] and by Keys et al. [25]

^b^Alleles with numbers greater than 100 contain an imperfect copy due to deletion or insertion and are reported instead as the lengths (in bp) of amplicons.

^c^Typability % = (number of isolates with PCR amplicon/number of isolates analyzed) × 100.

**Table 2:** Range of repeat number for 10 VNTR loci found in different serotype groups.

Locus	Serotype group:			
	
	1a/1b/NT (n = 12)	2a/2b/X/NT (n = 106)	3a/3b (n = 12)	4a/4b/Y (n = 110)
SF3	5-10	3-10	2-7	4-18
SF4	1-4	3-12	1-5	1
SF6	7-12	4-19	2	N
SF7	2	3-12	2	2
SF8	3	3-8	2	2
SF9	1-3	1-9	1	1-4
SF10	3	3-6	2	1-2
SF12	7	7	2-7	8, (389)^a^
SF25	2	2	4-11	1-2
SF31	2	2	2-11	2

^a^Number in the parenthesis denotes the amplicon size in base pairs for the allele.

### Phylogenetic relationships established using PFGE patterns

**Figure 1:**
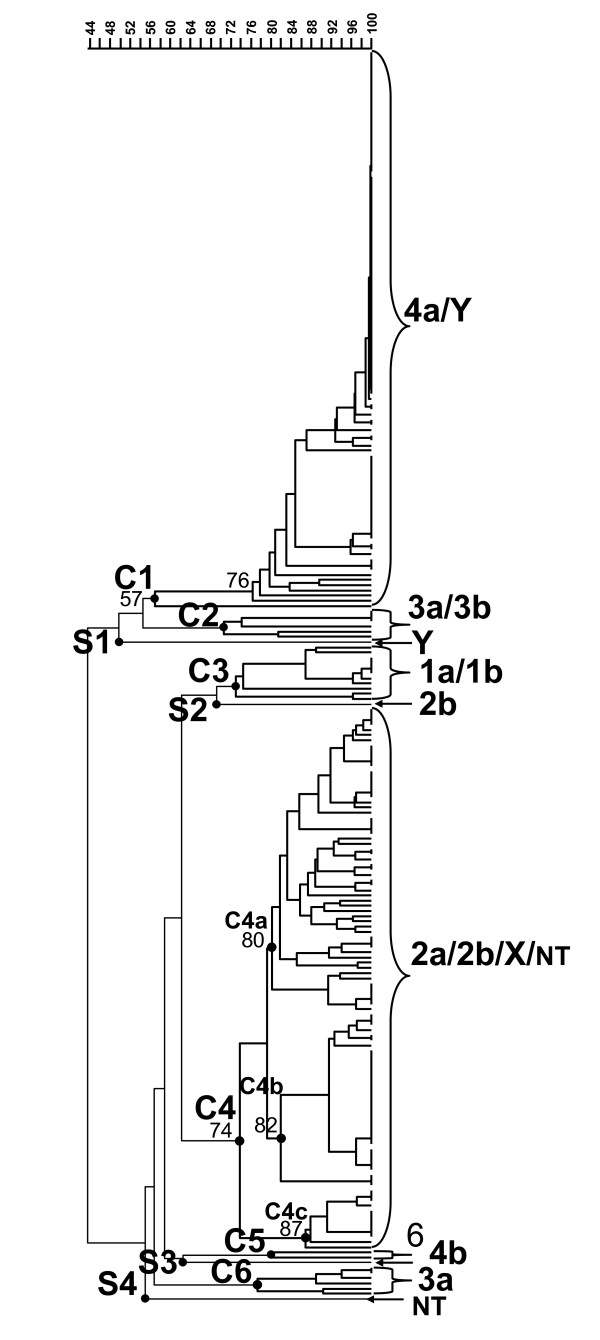
**Dendrogram for *Shigella flexneri *isolates**. The dendrogram is constructed using PFGE patterns for 242 *Shigella flexneri *isolates. Clusters are designated on the basis of the level of genetic relatedness and serotypes. Isolates in each of the three subclusters of cluster C4 shared at least 80% pattern similarity.

The PFGE analysis with NotI restriction enzyme identified 95 patterns among the 242 isolates. A dendrogram generated using the PFGE patterns is shown in Figure 1. The dendrogram with detail information including isolate code, PFGE code, serotype, year of isolation, MLVA code, outbreak and origin other than Taiwan is shown in Figure S1 (Additional file 2). Based on the serotypes and levels of genetic similarity among the isolates, six clusters (C1 to C6) and four singletons (S1 to S4) were designated. The groupings were correlated with serotypes. The clusters C1 to C6, respectively, contained isolates of 4a/Y, 3a/3b, 1a/1b, 2a/2b/X/NT, 6 and 3a. Isolates of serotype 3 were distributed in two distinct clusters, C2 (3a/3b) and C6 (3a). The four single singletons were respectively assigned for isolates with serotype Y, subserotype 2b, subserotype 4b, and NT. S2 (2b), originated from Egypt, was far distant from cluster C4 (2a/2b/X/NT). S4 (4b), originated from India, was also distantly related from cluster C1 (4a/Y). A serotype Y isolate and 102 subserotype 4a isolates within cluster C1 was recovered from a prolonged shigellosis outbreak occurring in a long stay psychiatric center [16]; they shared 76% or higher pattern similarity. These isolates shared only 57% similarity with another subserotype 4a isolate. The isolates within cluster C4 were further classified into three subclusters (C4a, C4b and C4c). Isolates within each of the three subclusters shared 80% or higher similarity. All the imported isolates in cluster C4 fell into subcluster C4a. Subcluster C4c consisted of 10 serotype X and two subserotype 2b isolates; the serotype X and one subserotype 2b isolates were recovered from a shigellosis outbreak.

### Phylogenetic relationships constructed using MLVA profiles

**Figure 2:**
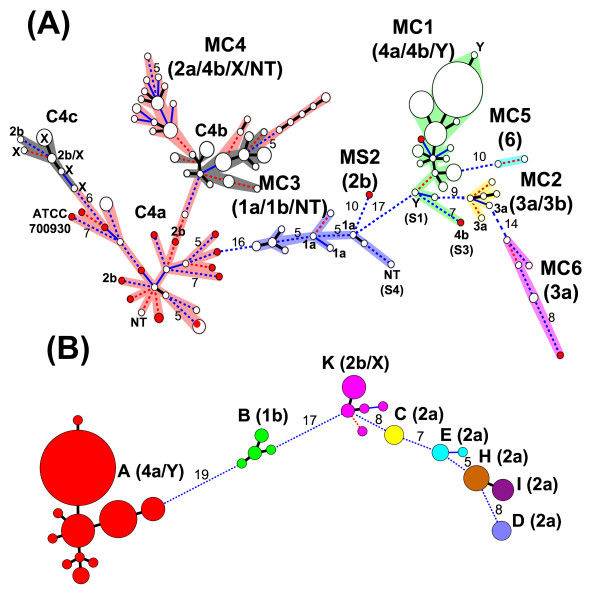
**Phylogenetic trees for *Shigella flexneri *isolates**. The phylogenetic trees are constructed with the MLVA profiles using an MST algorithm for (A) 242 isolates and for (B) 144 isolates from eight outbreaks. (A) Six clusters, each includes genotypes differing at eight loci or fewer among the 36 loci with the closest one, are designated and marked by different colors. The isolates in subclusters C4b and C4c defined by clustering of PFGE patterns are marked in grey. The singletons and the minor serotypes or subserotypes within a cluster are marked. (B) Outbreaks are indicated by different colors. Distances between outbreaks are numbered. A distance of one locus between two MLVA types is indicated by a thick line, a distance of two loci by a thin blue line, and a distance of three loci by a red broken line. Distances of four loci or greater are marked by a blue broken line. Distances between two genotypes differing at 5 or more loci are numbered. The area of the circle is proportional to the number of isolates belonging to the MLVA type.

In total, 104 MLVA types were identified in the 242 isolates. A phylogenetic tree was constructed using the MLVA profiles and a minimum spanning tree (MST) algorithm. The groupings established with MLVA profiles and PFGE patterns were highly concordant, with 83.8% congruence. On the basis of serotypes and the clusters established using PFGE data, a MLVA cluster was defined for genotypes that differed at eight loci or fewer among the 36 total loci. As a result, six MLVA clusters (MC1, MC2, MC3, MC4, MC5 and MC6) and one singleton (MS2) were designated (Figure 2A). The groupings based on PFGE and MLVA were almost consistent, except that the serotype Y (S1) and subserotype 4b (S3) isolates were grouped into cluster MC1 and the NT (S4) isolate was grouped into cluster MC3. Cluster MC1 consisted of 107 subserotype 4a, two serotype Y and one subserotype 4b isolates. One serotype Y and 106 subserotype 4a isolates were closely related; the serotype Y isolate differed at only one locus from the predominant type for subserotype 4a isolates. The remaining subserotype 4a isolate differed at two loci from a serotype Y (S1) isolate. The subserotype 4b (S3) had a distance of at least seven loci from others within cluster MC1. Cluster MC2 consisted of two subserotype 3a and four subserotype 3b isolates; they were closely related. One subserotype 3a isolate different at only two loci from a subserotype 3b isolate. Cluster MC3 contained three subserotype 1a, eight subserotype 1b and one NT isolates. They formed three tight subclusters; one subcluster consisted of six subserotype 1b isolates, which were recovered from an outbreak. Within this cluster, one subserotype 1a isolate differed at a single locus only from a subserotype 1b isolate. Cluster MC4 consisted of 10 serotype X, 90 subserotype 2a, four subserotype 2b and one NT isolates; they were relatively divergent. The three subclusters, C4a, C4b and C4c, designated on the basis of PFGE similarity were indicated in Figure 2A. The isolates in subcluster C4a showed a considerably diverse phylogenetic pattern; they were separated by subcluster C4b. The imported isolates were distributed more closely than most of others within this subcluster. The isolates in subcluster C4b as well as those in subcluster C4c were grouped tightly. Subcluster C4c consisted of 10 serotype X and two subserotype 2b isolates; one subserotype 2b isolate displayed an identical profile with a serotype X isolate. MC6 consisted of six subserotype 3a isolates; they formed a relatively diverse phylogenetic pattern. The imported isolate had a distance of at least eight loci from others within this cluster.

### Discriminatory power of PFGE and MLVA

PFGE, MLVA4 (an MLVA assay based on four most variable loci), MLVA8 and MLVA36 discriminated the total isolates into 95, 82, 90 and 104 genotypes, respectively (Table 3). More genotypes were obtained by PFGE than MLVA4 and MLVA8 but the level of discriminatory power for MLVA4 and MLVA8 was, though not significantly, higher than that for PFGE. For the panel of 90 diverse subserotype 2a isolates, MLVA4, MLVA8 and MLVA36 displayed better resolution than PFGE, though the difference of discriminatory power was not significant. PFGE, MLVA4 and MLVA36 divided the panel of 107 closely-related 4a/Y isolates into 19, 14 and 14 genotypes but the two MLVA assays exhibited higher discriminatory power than PFGE, though not significantly.

**Table 3:** The discriminatory index (DI) and 95% confidence interval (CI) of various typing methods for serotype groups.

Typing method	Total (n = 242)			2a (n = 90)			4a/Y (n = 107)^c^		
	
	No. types	DI	CI	No. types	DI	CI	No. types	DI	CI
PFGE	95	0.908	0.8778-0.9385	44	0.9421	0.9100-0.9742	19	0.5733	0.4681-0.6785
MLVA4^b^	82	0.9149	0.8879-0.9418	48	0.9745	0.9643-0.9847	14	0.6438	0.5503-0.7373
MLVA8^c^	90	0.9163	0.8891-0.9434	51	0.9778	0.9682-0.9874	NA	NA	NA
MLVA36	104	0.927	0.9008-0.9539	54	0.9800	0.9708-0.9893	14	0.6438	0.5503-0.7373

^a^Including an isolate with Y serotype, which was a single-locus variant of the predominant MLVA type of subserotype 4a.

^b^For the total: SF3, SF4, SF6, SF7; for 2a group: SF3, SF4, SF6, SF7; for 4a group: SF2, SF3, SF17, SF22

^c^For the total: SF3, SF4, SF6, SF7, SF8, SF9, SF10, SF25; 2a group: SF3, SF4, SF6, SF7, SF8, SF9, SF11, SF27

### Subtyping of isolates from outbreaks

A total of 144 isolates recovered from eight shigellosis outbreaks were used to evaluate the usefulness of MLVA in discriminating isolates for disease outbreak investigation. From outbreak A, 102 subserotype 4a and one serotype Y isolates were recovered (Table 4), while 10 serotype X and one subserotype 2b and were recovered from outbreak K. Isolates from four outbreaks (A, B, D and K) displayed two or more PFGE patterns, while the isolates from outbreaks H and I shared a common PFGE pattern.

**Table 4:** Characteristics of eight *Shigella flexneri *outbreaks.

Outbreak	Year	Serotype (no. isolates)	No. of PFGE types	No. of MLVA type	Locus difference
A	2001/10/8 - 2007/12/18	4a (102), Y (1)	15	11	SF2, SF3, SF17, SF22
B	2005/11/10 - 2005/11/11	1b (6)	4	4	SF3, SF26
C	2007/9/26 - 2007/10/27	2a (4)	1	1	
D	2008/2/15 - 2008/2/26	2a (4)	2	1	
E	2005/5/9 - 2005/11/4	2a (4)	1	2	SF3, SF6
H^a^	2005/5/19 - 2005/6/27	2a (7)	1	1	
I^a^	2005/10/22 - 2005/10/24	2a (5)	1	1	
K	2008/3/27 - 2008/4/4	X (10), 2b (1)	4	5	SF3, SF6, SF9, SF33

^a^Isolates for two outbreaks sharing a common PFGE pattern but different MLVA type

Isolates from four outbreaks (A, B, E and K) displayed two or more MLVA types (Figure 2B). The isolates from outbreaks H and I, sharing a common PFGE pattern, had different MLVA types. The isolates from four outbreaks (C, D, H and I) displayed only one MLVA type; notably, the isolates from these outbreaks were recovered over a short time period. The 103 isolates for outbreak A, recovered from a long-stay psychiatric nursing center over a long time period (2001-2007), were chronically divergent; four loci were variable among the isolates (Table 4). Outbreaks B and K, which occurred in a military camp and a long-stay psychiatric nursing center, respectively, lasted for only a few days; however, the isolates were considerably diverse. Four MLVA types were found in the six isolates from outbreak B, with variations in two loci. Five MLVA types were found in the 11 isolates from outbreak K, with variations in four loci; a distance of three loci was seen among the five types. The four isolates for outbreak E were recovered over a period of six months; the first three isolates shared the same MLVA type but the one recovered last differed at two loci from the first three.

## Discussion

VNTR markers display a wide range of variability; combining VNTR loci with various variability values can be used to discern various levels of genetic relatedness between bacterial isolates. In our previous studies, we identified 26 VNTR markers for *S. sonnei *and evaluated the usefulness of the MLVA method as a tool for fine typing and phylogenetic analysis of bacterial isolates [14,15]. These studies indicated that MLVA based on four to eight highly variable loci is sufficient to resolve closely related isolates for disease surveillance and investigations of outbreaks. In contrast, combining loci with lower variability values are suitable to establish clear phylogenetic patterns among strains evolved over a longer time span. Theoretically, the more number of loci are used, a higher discriminatory power can be achieved and subtler phylogenetic relationships among bacterial strains can be established. Unlike *S. sonnei, *which has only one serotype, *S. flexneri *has eight serotypes with at least 12 subserotypes [3,4]. Since VNTR markers can be serotype-specific, it is necessary to explore more loci at the moment of development.

In order to identify as many VNTR markers as possible, we explored VNTR candidates from the released genomic sequences of *S. flexneri *and other *Shigella *species using the VNTRDB computer program [17]. After testing 67 VNTR candidates on nine isolates of different subserotypes, we identified 36 loci that were polymorphic among the isolates. A further evaluation of the 36 loci on a total of 242 *S. flexneri *isolates from various serotypes and subserotypes suggested that variability of some of the 36 loci could be serotype-specific. For instance, seven loci (SF12, SF16, SF19, SF23, SF25, SF29, and SF31) were not polymorphic among the diverse isolates within the 2a/2b/X/NT group; but they were polymorphic among isolates in the 3a/3b, 4a/4b/Y and 6 groups (Table 1). In addition, SF25 and SF31 are highly variable in the 3a/3b group. However, this observation of serotype specificity could be biased because the number of isolates tested for a serotype group was limited or may have been clonal. For the 2a/2b/X/NT and 3a/3b groups, the isolates were relatively divergent and more variable loci were therefore observed. For the 1a/1b/NT, 4a/4b/Y and 6 serotype groups, the number of isolates was either too small or most isolates were closely related; only a small portion of the 36 loci was observed to be variable in these groups. It is expected that more variable loci could be found for the serotypes when a larger number of diverse isolates are analyzed.

Many studies have indicated that MLVA is more powerful than PFGE in discriminating bacterial isolates [12][14][18]; thus, it can complement or replace PFGE as a routine subtyping tool for outbreak investigation and disease surveillance. In the present study we showed that MLVA was able to discriminate isolates from two outbreaks that shared an indistinguishable PFGE pattern. For the panel of 90 diverse subserotype 2a isolates, the MLVA assays using four and eight most variable loci was able to discriminate the isolates into more genotypes than PFGE, although the discriminatory power was not significantly greater (Table 3). A similar result was observed on a panel of 107 closely related 4a/Y isolates. Although the MLVA assay using four most variable loci discriminated the 107 closely related 4a/Y isolates into fewer genotypes than PFGE, the discriminatory power for the MLVA4 assay was higher than that for PFGE. As the resolving power of MLVA to closely related isolates is primarily attributed to highly variable VNTR markers [15], an MLVA assay based on four to eight highly variable loci can display a resolving power comparable to or higher than PFGE.

VNTR markers have various degrees of variability, making MLVA as a useful molecular tool for phylogenetic study of bacterial isolates. MLVA has been applied to investigate the clonal relationships among isolates of *Yersinia pestis *[19], *N. meningitidis *[13,20] and *S. sonnei *[15]. In the present study, we used MLVA profiles to establish phylogenetic relationships among 242 *S. flexneri *isolates with various serotypes. By defining a clonal group as one that includes genotypes differing at eight loci or fewer between the two closest genotypes, the grouping based on the MLVA profiles is highly in agreement with that based on the PFGE patterns (Figure 1 and Figure 2A). The analysis based on MLVA profiles establishes clear phylogenetic patterns among different serotype groups. The isolates of serotype 3 are distributed into two distinct clonal groups, suggesting that the MLVA method developed in this study is also able to discern clonal groups among isolates within the same serotype.

The phylogenetic analysis using MLVA profiles as well as PFGE patterns also indicates that isolates with different subserotypes within a serotype can be genetically more closely related than those with the same subserotype. For example, one subserotype 1a isolate in cluster MC3 is more closely related to a subserotype 1b isolate than other subserotype 1a isolates (Figure 2A). A similar result is also observed between one subserotype 3a and one subserotype 3b isolates in cluster MC2. The analysis also indicates that the two serotype Y isolates and the 10 serotype X isolates could be derived from strains of serotype 4 and serotype 2, respectively, whereas the two NT isolates are related to serotype 1 and serotype 2.

Recently, Gorgé et al. [21] identified 15 VNTR loci by exploring five genomic sequences of four *Shigella *species for typing of *Shigella *spp. The 15 loci were evaluated on 89 isolates of *E. coli *and *Shigella *species including 19 *S. flexneri *isolates of eight serotypes. Thirteen of the 15 loci were polymorphic in the *S. flexneri *isolates. Of the 15 loci, 13 are included in the 36 loci identified in the present study. Although MLVA based on the 15 loci could effectively distinguish between the isolates of four *Shigella *species and pathogenic *E. coli*, grouping analysis based on the MLVA profiles was not able to distinguish among the four *Shigella *species. Although MLVA may not be effectively to establish phylogenetic relationships among isolates from different species of the same genus due to the specificity of VNTR markers; it can be a suitable phylogenetic analysis tool for monomorphic bacterial species, such as *S. sonnei *[15]. Our data indicate that the VNTR markers identified in the present study are applicable to establish clear phylogenetic relationships among serotypes groups and discerning clonal groups among isolates within a serotype.

A total of 144 isolates from eight outbreaks were analyzed using MLVA and PFGE in order to compare both genotyping methods in their ability to characterize isolates for disease outbreak investigation. MLVA was able to distinguish PFGE-indistinguishable isolates from two *S. flexneri *2a outbreaks. MLVA also discriminated isolates from four outbreaks into two or more genotypes. Theoretically, isolates collected from a prolonged outbreak, such as outbreak A, could have considerably been divergent, while isolates from an outbreak lasting for a short time period should have little variation. Strikingly, outbreaks B and K do not follow this pattern. These outbreaks started and ended within a few days but the isolates had showed considerably divergent. Several PFGE and MLVA genotypes were detected among the isolates for each of the outbreaks and no predominant type existed. This observation is difficult to explain. The strains could be hypervariable and mutants could emerge very quickly in response to host immunity, or the stains could have been circulating silently in the institutions for a while.

## Conclusions

A total of 36 VNTR markers were identified; they exhibited different degrees of variability among various *S. flexneri *serotype groups. VNTR locus could be highly variable in isolates of a serotype but invariable in other serotypes. The differences could be attributed to the fact that some of the loci were serotype-specific, the isolates for a serotype group analyzed were too clonal, or the number of isolates for a serotype group was limited. MLVA based on four highly variable loci is able to display a comparable resolving power to PFGE in discriminating isolates. MLVA is also a useful tool for phylogenetic analysis of *S. flexneri*; it can establish clear clonal patterns among serotype groups and also discerned clonal groups among isolates within a serotype, for example serotype 3. Isolates with different subserotypes within a serotype can be genetically more closely related than those with the same subserotype. Isolates that had different serotype but had closer genetic relatedness than those with the same serotype were observed between serotype Y and subserotype 4a, serotype X and subserotype 2b, subserotype 1a and 1b, and subserotype 3a and 3b. As highly variable VNTR loci could be serotype-specific, a common MLVA protocol that consists of four to eight loci and that provides high resolving power to all *S. flexneri *serotypes may not be obtainable.

## Methods

### Bacterial strains

A total of 241 *S. flexneri *isolates were collected in Taiwan between 1995 and 2008, including serotypes and subserotypes 1a (3 isolates), 1b (8), 2a (89), 2b (5), 3a (8), 3b (4), 4a (107), 4b (1), serotype X (10), Y variant (2), 6 (2) and non-typable (NT) (2). One reference strain, *S. flexneri *2a strain 2457T (ATCC 700930), was purchased from the ATCC Global Bioresource Center. Among the isolates, 18 were recovered from patients infected during the travelling to Cambodia (4 isolates), China (6), Egypt (1), India (3) and Indonesia (4), and 144 were recovered from eight shigellosis outbreaks. Of the 107 subserotype 4a isolates, 102 were collected from a prolonged shigellosis outbreak that occurred in 2001-2007 in a long-stay psychiatric nursing center [16]; they were expected to be closely related. In contrast, the serotype 2a isolates were chronologically divergent, even though 24 isolates were recovered from five shigellosis outbreaks.

### PFGE

The PulseNet PFGE protocol for *S. sonnei *and other enterobacteria was used for PFGE analysis [22] except that 5 U of *Not*I instead of *Xba*I was used for the restriction digestion.

### Identification of VNTR loci

Firstly, the three genomes of *S. flexneri *serotype 2a strain 301 (GenBank accession no. AE005674), *S. flexneri *serotype 2a strain 2457T (GenBank accession no. AE014073) and *S. flexneri *serotype 5 strain 8401 (accession no. CP000266) were explored for potential VNTR loci using VNTRDB computer software developed by Chang et al. [17]. As a result, 50 loci were selected for further evaluation. In order to maximize the number of VNTR markers for *S. flexneri*, another search on five genomes of three *Shigella *species, including *S. dysenteriae *strain Sd197 (accession no. CP000034), *S. flexneri *serotype 2a strains 301and 2457T, *S. sonnei *strains Ss046 (accession no. CP000038) and 53G (Wellcome Trust Sanger Institute http://www.sanger.ac.uk), was performed to explore potential VNTR loci. Consequently, 17 additional loci were selected for further evaluation. In total, 67 loci were examined for their variability within nine *S. flexneri *isolates of various subserotypes by PCR amplification. To perform PCR reactions, primers for the 67 loci were designed using the Primer3 program available on the website, http://frodo.wi.mit.edu. PCR reactions were carried out in a GeneAmp PCR System 9600 (Applied BioSystems). For PCR amplification, crude bacterial DNA was prepared by the boiling method as described [14]. Each 10-μl PCR mixture contained 1x PCR buffer, 3 mM MgCl_2_, 0.2 μM of each primer, 200 μM of each deoxyribonucleotide, 1.0 unit of the recombinant SuperNew Taq DNA polymerase (Jier Sheng Company, Taipei, Taiwan), and 1 μl of DNA template. The PCR reaction was performed with a denaturing step at 94°C for 5 min, followed by 30 cycles of amplification at 94°C for 30 s, 55°C for 45 s, and 72°C for 45 s, and extended at 72°C for 5 min at the final step. Loci with varied sizes among the nine isolates were considered to be VNTR loci and the markers were used to further analyze a total of 242 *S. flexneri *isolates.

### MLVA

The primer sets for PCR amplification of 36 VNTR loci are listed in Table S2 (Additional file 3). The forward primer for each primer set was labelled at its 5' end with an ABI-compatible dye, 6-FAM, NED, VIC or PET by Applied BioSystems (Foster City, CA, USA). Nine multiplex PCR combinations were set for the analysis. PCR reactions were performed as described above except that dye-labelled primers were used. Occasionally, no amplicon was detected for some loci by multiplex PCR; in these cases, the loci were amplified individually. If no amplicon was detected by amplification individually, DNA of the isolates prepared by a commercial kit (Geneaid, Taipei County, Taiwan) was used for amplification. The PCR products were analyzed by capillary electrophoresis on an ABI Prism 3130 Genetic Analyzer with GeneScan 500 LIZ Size Standard (cat # 4322682; Applied BioSystems) as described [14].

### Data analysis

PFGE images were analyzed using the fingerprint analysis software BioNumerics version 4.5 (Applied Maths; Kortrijk, Belgium). A PFGE genotype was defined as a PFGE pattern with one or more DNA bands different from the others. A dendrogram constructed using the *Not*I-digested PFGE patterns was generated by the UPGMA algorithm with the Dice-predicted similarity value of two patterns at 1.0% pattern optimization and 0.8% band position tolerance. The number of repeat units for each allele was converted from the length of amplicon, saved as "Character Type" in the BioNumerics database, and subjected to cluster analysis using the MST algorithm and categorical coefficient provided in the BioNumerics software. Creation of hypothetical types (missing links) was allowed to introduce hypothetical types, group minimum size was set at 2 and maximum neighbour distance was set at 8. Alleles, which contained imperfect repeat unit(s) due to deletion or insertion, were designated by the lengths (in bp) of amplicons. To compare the discriminatory power of PFGE and MLVA with various combinations of VNTR loci, Simpson's index of diversity (*D*) and 95% confidence intervals (CI) were calculated according to the formulas as the described [23,24]. The polymorphism of each locus was represented by Nei's diversity index, calculated as 1-∑ (allelic frequency)^2^.

## Authors' contributions

CS Chiou initiated and managed the project, analyzed data and wrote the manuscript. YW Wang worked on MLVA, PFGE and data analysis. H Watanabe, DC Phung and J Terajima shared the work on the management of the project and analyzing and interpreting data. YS Lee helped to collect isolates and epidemiological data. SK Tung explored potential VNTR loci from genomic sequences. SY Liang shared a part of MLVA work. All the authors have read and approved the final manuscript.

## Supplementary Material

Additional file 1 **Supplementary Table S1. Characteristics of 36 VNTR loci in three *Shigella flexneri *strains**. This file can be viewed with: Microsoft Excel Viewer. Click here for file

Additional file 2 **Supplementary Figure S1. Dendrogram generated using the PFGE patterns**. This file can be viewed with: Adobe Acrobat Reader. Click here for file

Additional file 3 **Supplementary Table S2. Primers, dyes and multiplex PCR combinations**. This file can be viewed with: Microsoft Excel Viewer. Click here for file
