# Supplementary material for: Multilocus variable-number tandem repeat analysis for molecular typing and phylogenetic analysis of Shigella flexneri
Source: BMC Microbiol. 2009 Dec 31;9:278. doi: 10.1186/1471-2180-9-278 (PMC2806262; doi:10.1186/1471-2180-9-278)
Supplement: Additional file 2 — Supplementary Figure S1. Dendrogram generated using the PFGE patterns. This file can be viewed with: Adobe Acrobat Reader. [file 1471-2180-9-278-S2.PDF]

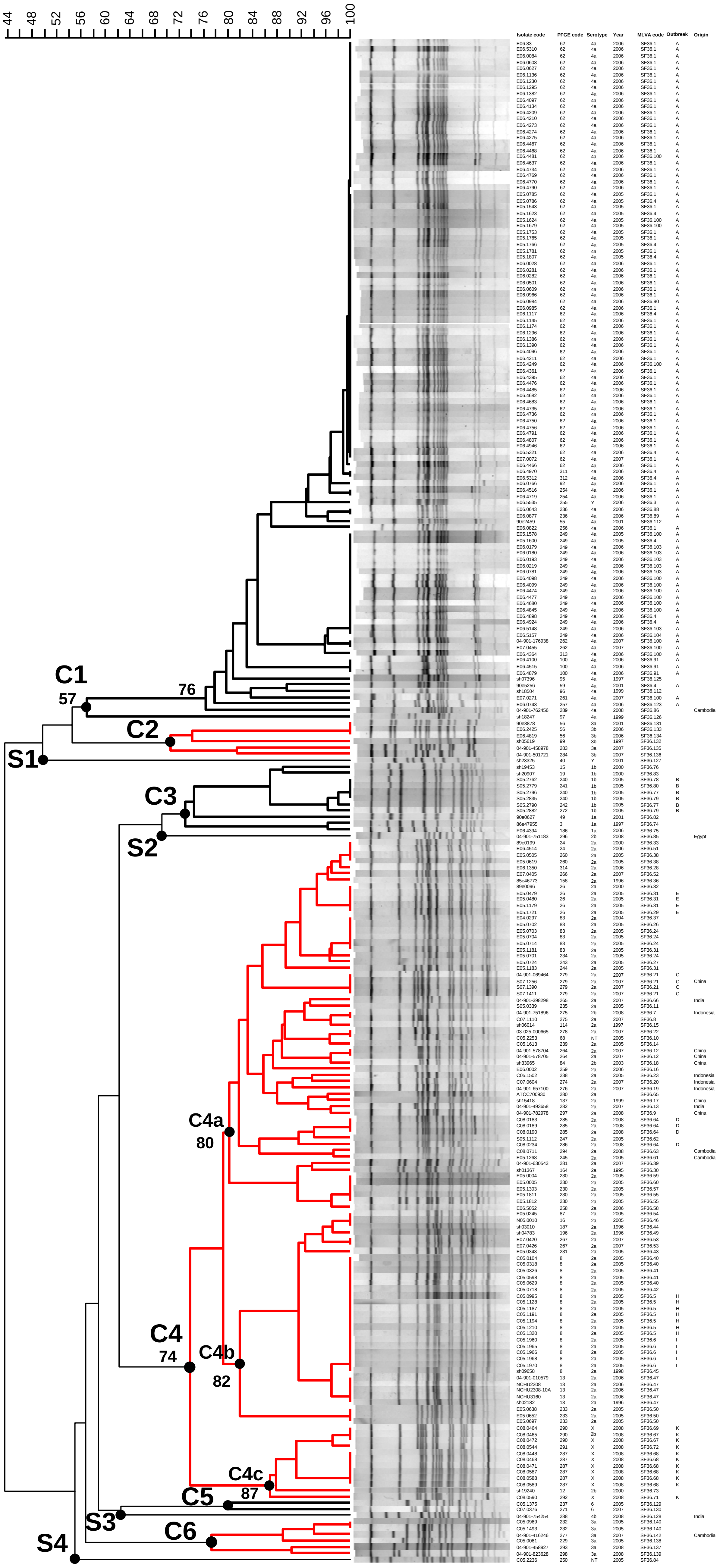

44
48
52
56
60
64
68
72
76
80
84
88
92
96
100
Isolate code
PFGE code
Serotype
Year
MLVA code
Outbreak
Origin
C1
57
76
C2
S1
C3
S2
C4a
80
C4
74
C4b
82
C4c
87
C5
S3
C6
S4
